# Concordance Between Watson for Oncology and Multidisciplinary Teams in Colorectal Cancer: Prognostic Implications and Predicting Concordance

**DOI:** 10.3389/fonc.2020.595565

**Published:** 2020-12-23

**Authors:** Chenchen Mao, Xinxin Yang, Ce Zhu, Jingxuan Xu, Yaojun Yu, Xian Shen, Yingpeng Huang

**Affiliations:** Department of Gastrointestinal Surgery, The Second Affiliated Hospital of Wenzhou Medical University, Wenzhou, Zhejiang, China

**Keywords:** Watson for oncology, multidisciplinary teams, colorectal neoplasms, concordance, prognosis, nomogram

## Abstract

**Background:**

Watson for Oncology (WFO) is a cognitive computing system that provides clinical decision support. This study examined the concordance between the treatment recommendations for colorectal cancer (CRC) proposed by WFO and those recommended by the multidisciplinary teams (MDTs), and evaluated the influence of concordance on the prognosis.

**Methods:**

We retrospectively collected 175 patients with colorectal cancer who received treatment recommended by MDTs at a hospital in China, and evaluated them using WFO. Concordance between the two recommendations was analyzed. The overall survival was analyzed between concordant and non-concordant groups. Logistic regression analyses were performed and a concordance-predicting model was developed.

**Results:**

Concordance between WFO’ and MDTs’ recommendations occurred in 66.9% (117/175) of cases. The overall survival (OS) was significantly better in concordant group and non-concordance was found to be an independent prognostic factor [hazard ratio (HR)=2.784 (95% CI 1.264–6.315)]. Logistic regression analyses determined that tumor type [odds ratio (OR)= 2.195 for left colon cancer and OR=2.502 for rectum cancer], and TNM stage (OR=0.545 for stage II, OR=0.187 for stage III, OR=0.127 for stage IV) were independently related with concordance, which were used to develop a concordance-predictive-nomogram.

**Conclusions:**

Treatment recommendations for patients with colorectal cancer determined by WFO and MDTs were mostly concordant. However, the survival was better among concordant patients and non-concordance was found to be an independent prognostic factor. This study presents a nomogram that can be conveniently used for predicting individualized concordance. However, our findings should be prospectively validated in multi-center trials.

## Introduction

Colorectal cancer (CRC) is the third most common cancer in males, and the second most common cancer in females according to global cancer statistics (1). Presently, chemotherapy options for patients with colorectal cancer are determined by multidisciplinary teams (MDTs), based on the National Comprehensive Cancer Network (NCCN) guidelines, combined with clinical experience and findings of recent studies. (2, 3) However, compared with almost 2.8 physicians for every 1,000 individuals in the United States and other developed countries, there are only 1.2 physicians for every 1,000 individuals in China (4, 5). Conversely, while the medical data, papers, and guidelines in tumor-related fields are rapidly growing, the time that practitioners can devote to learning is limited. A study showed that oncologists spend approximately 4.6 hours per week to enhance professional knowledge (6). Therefore, considering the contradiction between the need for individualized treatment plans for every patient, and the greatly imbalanced distribution of medical resources, as well as the inconvenience of organizing MDT discussions, a tool that can assist practitioners to quickly provide accurate treatment recommendations and learn the new developments of the field in a more efficient manner is urgently needed.

Recently, artificial intelligence (AI) is being increasingly used to support the field of medicine; computational analysis tools, and decision support systems can help with disease diagnosis, and selecting appropriate therapeutic procedures (7). Notably, three clinical decision support systems (CDSS)—Clinical Oncology’s Cancer Linq, Oncodoc, and International Business Machines (IBM) Watson for Oncology (WFO)—have been used in medical oncology (8, 9). Of these, WFO recommends treatment options based on the literature, protocols, and the patient’s chart, in addition to the experiences from prior cases and experts at the Memorial Sloan Kettering Cancer Center (MSKCC) (10). Somashekhar et al. (10) reported that the treatment concordance between WFO and multidisciplinary tumor board occurred in 93% of 638 breast cancer cases, suggesting that the AI clinical decision support system may be a helpful tool for treatment-related decision-making in breast cancer. Zhou et al. (11) found that WFO might be useful in recommending postoperative therapy for GI tract tumors with the concordance of 74, 64, and 12% for rectal cancer, colon cancer, and gastric cancer respectively. However, most studies focused on the overall concordance, and neglected the individual usability of WFO. In addition, the researches on colorectal cancer have been limited, so far.

Therefore, we aimed to assess the concordance between WFO recommended treatments and the actual therapeutic regimens that were determined by MDTs in our cancer center for patients with colorectal cancer and compare the patient prognosis between those with and without this concordance. Moreover, we aimed to develop and validated a nomogram that incorporated the clinicopathologic risk factors for individualized prediction of concordance.

## Materials and Methods

### Study Population

**Figure 1:**
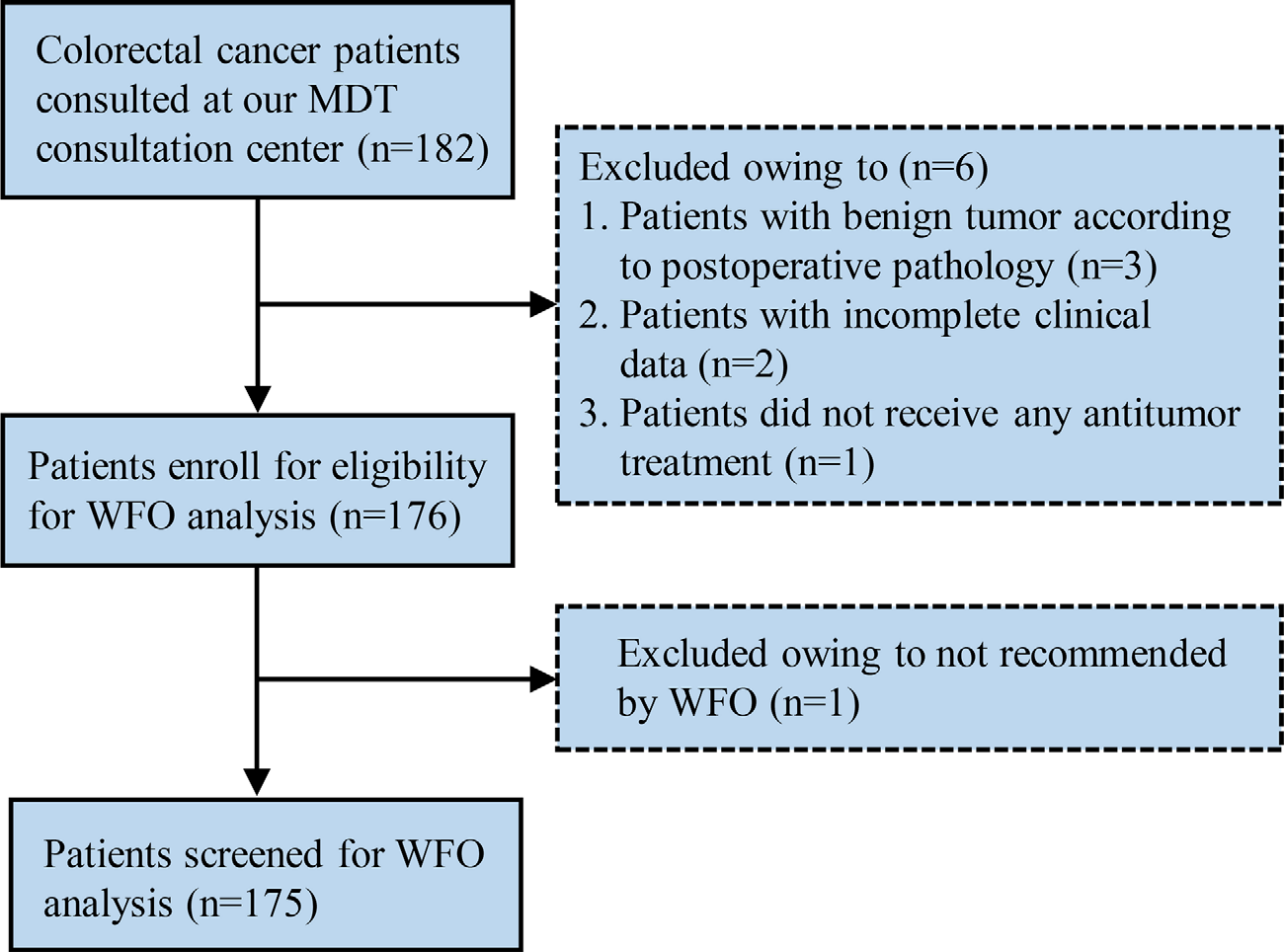
Flow diagram of the patient selection process. MDT, multidisciplinary team; WFO, Watson for Oncology.

In this retrospective study, the data of 182 patients with colorectal cancer treated between January 2016 and January 2018 at the Gastrointestinal Surgical Departments of the Second Affiliated Hospital of Wenzhou Medical University were randomly selected. Additionally, each patient’s therapy was determined by MDTs, including, but not limited to, specialists from the departments of gastrointestinal surgery, oncology surgery, gastroenterology, radiotherapy and chemotherapy, and radiography. Patients with benign tumors according to postoperative pathology, those with incomplete clinical data, and those who did not receive any antitumor treatment were excluded. Detailed flow diagram of patient selection in this study is shown in **Figure 1**. The study was approved by the ethics committees of the Second Affiliated Hospital of Wenzhou Medical University, and all participants provided written informed consent prior to study participation, in accordance with the tenets of the Declaration of Helsinki.

### Watson for Oncology

The patients’ clinicopathologic data were collected and logged into the WFO system by two senior physicians who were blinded to the actual therapy. WFO provided therapeutic recommendations in three categories: recommended, for consideration, and not recommended. Additionally, we defined it “physician’s decision” when actual therapeutic regimens were not available in WFO. Data were further analyzed to compare the WFO’s recommendations and actual therapeutic regimens used in our hospital. Actual therapeutic regimens were considered as concordant with WFO if they corresponded to the “recommended” or “for consideration” categories, otherwise, they were defined as non-concordant.

### Data Analysis and Statistics

The probabilities of overall survival (OS) and disease-free survival (DFS) were estimated by using the Kaplan–Meier method and evaluated *via* log-rank test. The Cox proportional hazard model was used to estimate the risk ratio in univariate, and multivariate analyses. To control the determinants of concordance, univariate logistic regression analysis was performed first. Then, based on the clinical predictors that were statistically significant in univariate analysis, multivariable logistic regression analysis employing forward step-wise selection was performed. A nomogram that could quantitatively predict concordance probability between WFO’s recommendations and actual therapeutic regimens was constructed based on the results of multivariable logistic analysis. Decision curve analysis (DCA) and receiver operating characteristic (ROC) curve were conducted to evaluate the clinical usefulness and accuracy of the nomogram, respectively. All *P*-values were two-sided, and *P*<0.05 was considered statistically significant. All statistical analyses were performed using SPSS software (version 22.0; SPSS Inc., Chicago, IL, USA) and R software (version 3.0.1; http://www.Rproject.org).

## Results

### Baseline Characteristics of Patients

Of the 182 eligible patients, 175 were finally recruited for the study. The baseline clinicopathological characteristics of the patients are detailed in **Table 1**. Among them, 24.6% (43/175) patients had right colon cancer, 32.6% (57/175) had left colon cancer, and 41.7% (73/175) had rectal cancer. A majority of the patients were male (n=109, 62.3%) and older than 60 years (n=115, 65.7%). Additionally, patients with large tumor (>5cm), poorly differentiated, Ulcerative type, and TNM stage II/III accounted for 44.0% (77/175), 80.6% (141/175), 76.6% (134/175), and 74.8% (73 + 58/175), respectively.

**Table 1:** Demographics and characteristics of patients using Watson for Oncology.

Factors	Total (n = 175)
Gender	
Male	109 (62.3%)
Female	66 (37.7%)
Age (y)	
<60	60 (34.3%)
≥60	115 (65.7%)
Mean ± SD	63.83 ± 12.57
BMI (kg/m2)	
18.5-24	88 (50.3%)
<18.5	22 (12.6%)
≥24	50 (28.6%)
Mean ± SD	22.49 ± 3.50
Tumor type	
Right colon cancer	43 (24.6%)
Left colon cancer	57 (32.6%)
Rectal cancer	73 (41.7%)
Tumor size (cm)	
<5	95 (54.3%)
≥5	77 (44.0%)
Mean ± SD	4.64 ± 2.00
Histopathological differentiation	
Poor-differentiated	141 (80.6%)
High-differentiated	28 (16.0%)
Pathologic type	
Ulcerative type	134 (76.6%)
Non-ulcerative type	37 (21.1%)
TNM stage	
Stage I	24 (13.7%)
Stage II	73 (41.7%)
Stage III	58 (33.1%)
Stage IV	17 (9.7%)

Data are presented as n (%), mean ± SD unless otherwise indicated.

BMI, body mass index.

*Statistically significant (P< 0.05).

### Concordance Between WFO’ and MDTs’ Recommendations

**Figure 2:**
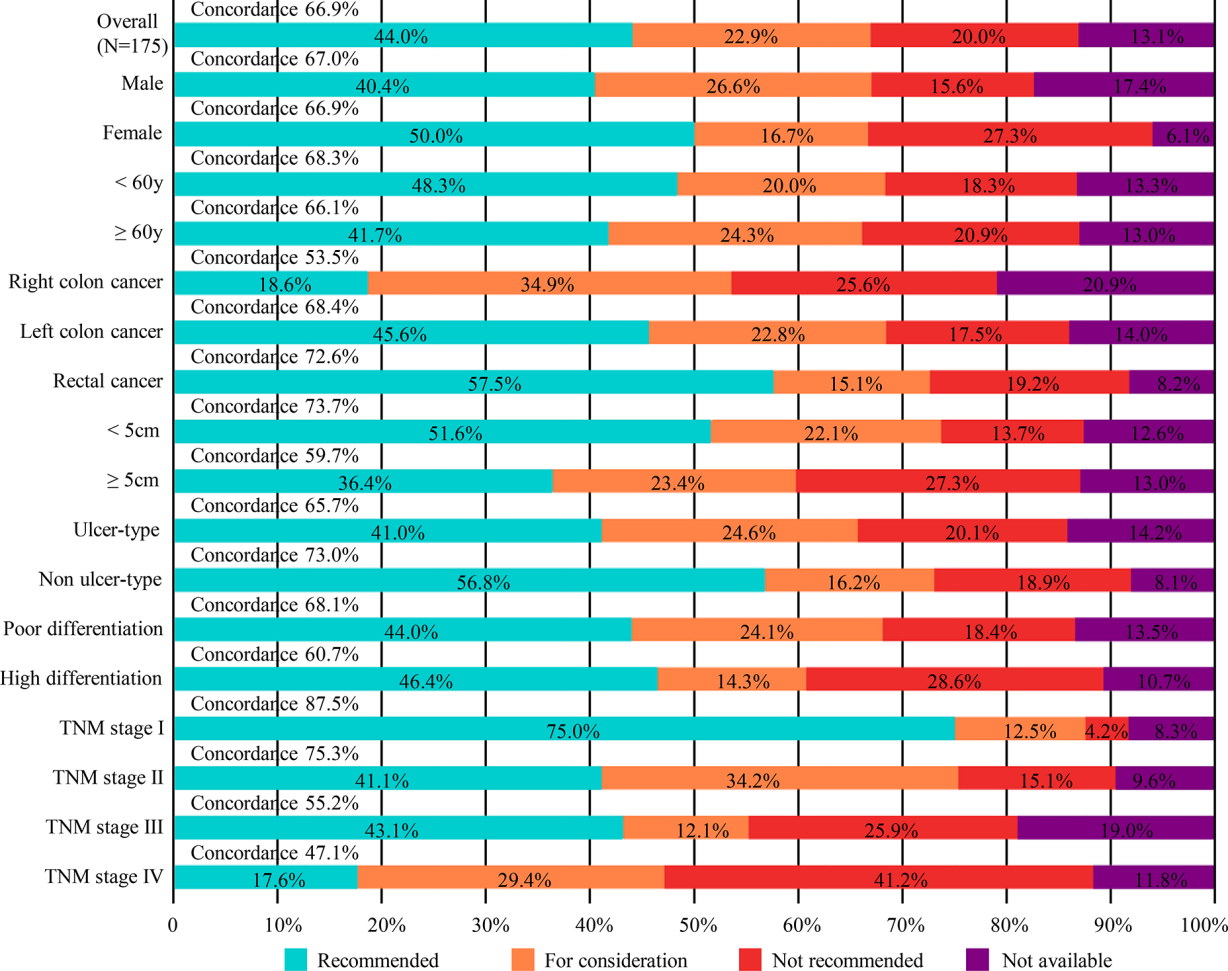
Treatment concordance between WFO and MDT decision, divided by gender, age, tumor type, tumor size, pathologic type, histopathological differentiation and TNM stage. MDT, multidisciplinary team; WFO, Watson for Oncology.

When comparing the treatment recommendations of MDTs and WFO, treatment options that were designated as “recommended”, “for consideration”, “not recommended”, and “physician’s decision” accounted for 44.0% (77/175), 22.9% (40/175), 20.0% (35/175), and 13.1% (23/175), respectively (**Figure 2**). Of the 175 patients analyzed, the treatment recommendations were concordant in 66.9% (117/175). Subgroup analysis of therapy concordance with clinicopathological characteristics showed that patients with left colon cancer/rectal cancer [68.4% (39/57), 72.6% (53/73), respectively], small tumor (<5cm) [73.7% (70/95)], non-ulcer type cancer [73.0% (27/37)], poorly differentiated tumor [68.1% (96/141)], and TNM stage I/II [87.5% (21/24), 75.3% (55/73), respectively] exhibited higher concordance than those with right colon cancer [53.4% (23/43)], large tumor (≥5cm) [59.7% (46/77)], ulcer type cancer [65.7% (88/134)], highly differentiated tumor [60.7% (17/28)], and TNM stage III/IV disease [55.2% (32/58), 47.1% (8/17), respectively]. While, no obvious difference was found between males and females as well as old and young groups.

### Prognostic Analysis

**Figure 3:**
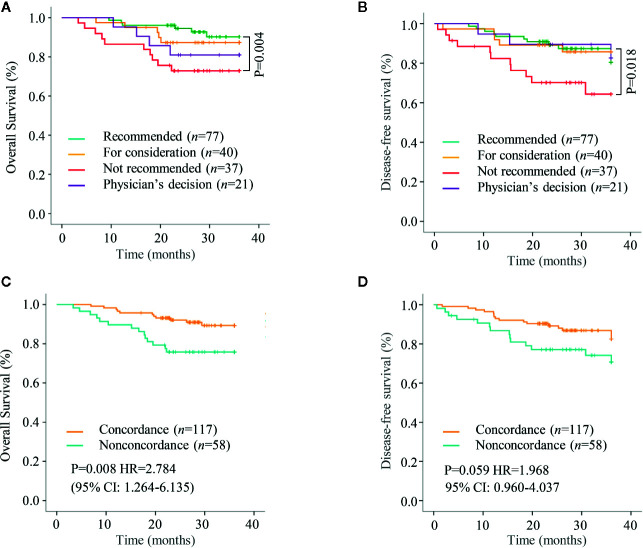
Kaplan–Meier curves for overall survival (OS) and disease free survival (DFS). **(A, B)** Overall survival and Disease free survival was analyzed and compared between patients with different treatment options groups. **(C, D)** Overall survival and Disease free survival were analyzed and compared between the concordant group and the non-concordant group.

In Kaplan–Meier’s analyses for different treatment options groups, the overall survival of “recommended” group was better than “not recommended” group (log-rank test P=0.004; **Figure 3A**), while no other significant difference was found in the survival curve. Similar results were noted for disease-free survival (log-rank test P=0.018; **Figure 3B**).

While analyzing the differences between the concordant group and the non-concordant group, the overall survival was better in concordant group than that in the non-concordant group (log-rank test p=0.008; **Figure 3C**). Further, Cox regression analyses determined non-concordance (HR=2.784; 95% CI: 1.264–6.135) as an independent risk factor for overall survival (**Figure 3C**). Although the concordant group had a better disease free survival than the non-concordant group, however, no statistical significance was reached (log-rank test P=0.059; **Figure 3D**).

### Univariate and Multivariate Analysis of Variables Associated With Concordance

Univariate and multivariate logistic regression analyses were performed to examine the variables associated with concordance between WFO’ and MDTs’ recommendations. As shown in **Table 2**, tumor type (*P*=0.130, for left colon cancer; *P*= 0.038, for rectal cancer), and TNM stage (*P*=0.219, for stage II; *P*=0.010, for stage III; *P*=0.009, for stage IV) were significantly correlated with concordance. Further multivariate logistic regression analysis identified that both, the tumor type (odds ratio (OR) 2.195, 95% confidence interval (CI) 0.911–5.291, *P*=0.080, for left colon cancer; and OR 2.502, 95% CI 1.061–5.881, *P*=0.036, for rectal cancer), and TNM stage (OR 0.545, 95% CI 0.141–2.106, *P*=0.379, for stage II; OR 0.187, 95% CI 0.050–0.707, *P*=0.013, for stage III; and OR 0.127, 95% CI 0.031–0.711, *P*=0.017, for stage IV) as independent predictors.

**Table 2:** Logistic regression model of concordance between WFO and MDT.

Factors	Univariate		Multivariate	
	HR (95% CI)	*P* value	HR (95% CI)	*P* value
Gender				
Male	Reference			
Female	1.014 (0.530–1.940)	0.967		
Age (y)				
<60	Reference			
≥60	1.107 (0.568–2.158)	0.764		
BMI (kg/m^2^)				
18.5-24	Reference			
<18.5	1.745 (0.663–4.593)	0.260		
≥24	1.417 (0.676–2.972)	0.356		
Tumor size (cm)				
<5	Reference			
≥5	0.530 (0.278–1.010)	0.054		
Tumor type				
Right colon cancer	Reference		Reference	
Left colon cancer	0.531 (0.234–1.204)	0.130	0.456 (0.189–1.098)	0.080
Rectal cancer	0.434 (0.197–0.956)	0.038	0.400 (0.170–0.940)	0.036
Histopathological differentiation				
High-differentiated	Reference			
Middle low-differentiated	0.724 (0.314–1.673)	0.450		
Pathologic type				
Ulcerative type	Reference			
Non-ulcerative type	0.709 (0.316–1.590)	0.404		
TNM stage				
Stage I	Reference		Reference	
Stage II	2.291 (0.611–8.590)	0.219	1.833 (0.475–7.079)	0.379
Stage III	5.687 (1.526–21.200)	0.010	5.341 (1.415–20.156)	0.013
Stage IV	7.875 (1.689–36.720)	0.009	6.695 (1.406–31.881)	0.017

Data are presented as median (IQR).

*Statistically significant (P < 0.05).

### Development of an Individualized Prediction Model

**Figure 4:**
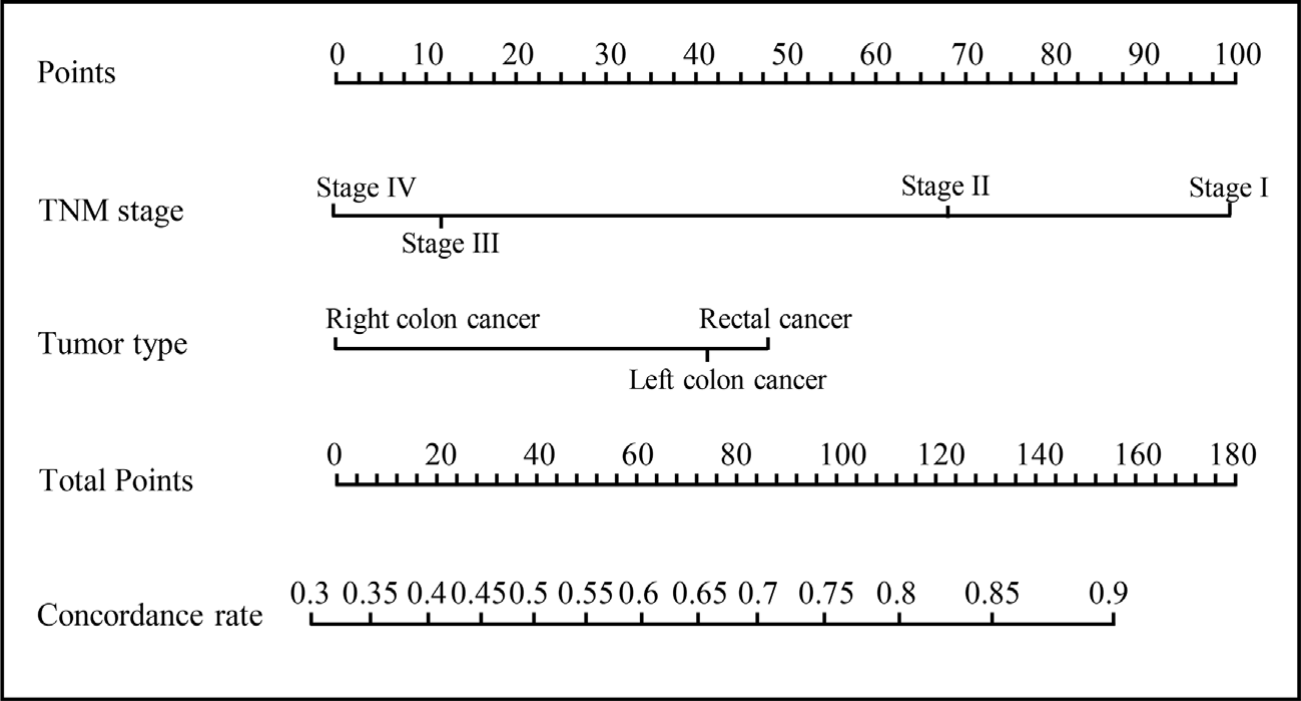
Developed nomogram. The nomogram was developed with the TNM stage and tumor type.

**Figure 5:**
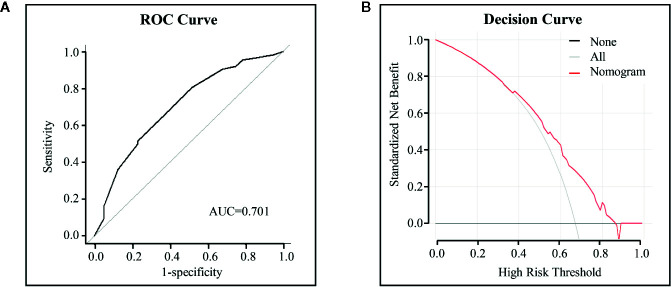
ROC curves and decision curve analysis for the nomogram. **(A)** ROC curves to identify concordance. The area under the ROC curve (AUC) values were shown. **(B)** Decision curve analysis for the nomogram. The y-axis measures the net benefit. The red line represents the nomogram. The gray line represents the assumption that all cases were concordant and the black line represents the assumption that no cases were concordant.

A model that incorporated the above independent predictors was developed and presented as a nomogram (**Figure 4**), with a C-index of 0.700. The ROC curve (**Figure 5A**) for the nomogram demonstrated that the nomogram had a high predictive accuracy for concordant rate [area under the curve (AUC)=0.702]. Additionally, decision curve (**Figure 5B**) showed that, if the threshold concordance probability of a patient was 33–86%, using the nomogram to predict concordance, and treat the patient as WFO recommended, would add greater benefit than would be achieved by either treating all, or none of the patients according to WFO recommendations. For example, if the personal threshold probability of a patient was 60% (i.e., the patient would opt for therapeutic regimens recommended by WFO if the probability of concordance was 60%), then the net benefit would be 0.383 if the nomogram was used to decide whether to treat as WFO recommended, with added benefits over using WFO for either all, or none of the patients.

## Discussion

To the best of our knowledge, this is the first study that examined the concordance between the treatment regimens used by MDTs and those recommended by WFO as well as the survival impact of concordance in patients with colorectal cancer.

We found that the overall concordance between the therapeutic recommendations of WFO and the regimens used by MDTs were 66.9%. Although it was obviously lower than that reported from Korea by Kim et al. for colorectal cancer, (3) it was similar to the concordance of 64 and 74% for colon cancer and rectal cancer, respectively in another study (11). Among the non-concordant cases, several of those resulted from aggressive treatment approaches, or the forgoing of chemotherapy based on demographic characteristics such as comorbidity burden, patient preferences, and level of social support systems. However, these data can be adjusted by including patient income levels and medical security types in the WFO system, and a more appropriate treatment can be recommended. Additionally, there were a large proportion of patients who received “physician’s decision” therapy that was not available in WFO because of the imperfection of the WFO system of lacking recommended treatment of neoadjuvant chemotherapy, and chemotherapeutic drugs such as docetaxel, irinotecan, and PD-1/PD-L1 antibodies, however, this can be adjusted with the update and further development of WFO system. Additionally, among the 25 (13. 1%) patients received “physician’s decision” therapy, 13 patients received excessive chemotherapy including 7 of them were treated with targeted chemotherapy drugs in addition to the recommended regimen while 6 patients received systemic chemotherapy although the WFO recommendation was “Surveillance”. Additionally, comprehensively considering the patient’s condition and family members’ willingness to treat, there are also some patients received palliative surgery according to MDT. It is worth mentioning that the concordance rate decreased with the increase of TNM stage, considering the difference between the disease and the patient’s financial situation, targeted chemotherapy drugs or palliative surgery may both recommended for patients with TNM stage IV. It also reflects the improvement of WFO system for consideration of targeted recommendation scheme as well as the actual patients’ economic situation.

In this study, we first analyzed the relationship between concordance and survival. We found that the OS and DFS correspondingly decreased with the reduction of recommendation level when we divided the patients into “recommended” group, “for consideration” group, “not recommended” group, and “physician’s decision” group. However, the statistical significance was only noted between the “recommended” group and “not recommended” group because of our small sample size, and limited follow-up time, which to some extent proved the effectiveness of WFO system to aid toward achieving a good prognosis. Additionally, similar to a recent study (12) that demonstrated that the overall survival of patients with gastric cancer in the concordant group was better than that in the non-concordant group, we further found that both, OS, and DFS were better in the concordant patients, although no statistical significance was reached for DFS. That also greatly attributed to the better prognosis of the 13 over treated patients in “physician’s decision” group. As for the remaining patients who received neoadjuvant chemotherapy, immunotherapy, and radical chemotherapy in “physician’s decision” group, most of them had complex conditions, thus MDT recommended comprehensive therapy was still needed. In general, we believe that WFO system could provide a great assistance to the MDT, as it provides treatment advices based on the updated knowledge and comprehensive evidence.

It is worth mentioning that unlike other studies that only focused on consistency rate (3, 10, 11, 13), a nomogram, which incorporated tumor type, and TNM stage, could indicate individual concordance possibilities between WFO and MDT recommendations with high sensitivity and specificity was first developed in the current study. Although we attempted to subdivide the patients by tumor type, and develop nomograms separately; however, we were unsuccessful because of the small sample size (data not shown). Further, TNM stage was another independent risk factor consistent with previous studies (10, 14). Patients treated with the same chemotherapy regimens as determined by MDTs, and WFO tended to have earlier tumor stages, which might be attributable to the socioeconomic characteristics of the patients. Patients with later TNM stage disease might have opted to abandon the treatment or choose cheaper and more conservative chemotherapy because they could not afford postoperative chemotherapy, or they might have chosen more radical chemotherapy according to their economic capacity. The most important argument for the use of the nomogram is based on the adoption of individualized therapeutic regimens recommended by WFO. With this aim, decision curve analysis, which offers insight into clinical consequences on the basis of threshold probability from which the net benefit could be derived, was applied in this study. The decision curve showed that, if the threshold probability of a patient determined by the nomogram in the current study was more than 33%, choosing the WFO-recommended chemotherapy regimens would add greater benefit than would either treating all or none of the patients as recommended by WFO, in the absence of the ability to organize discussion among MDTs.

There are some limitations in this study. First, this was a retrospective study with a small sample size, the baseline differences between the groups and some subgroups could not be eliminated; a randomized clinical trial with large sample is thus needed in future. Second, the follow-up time in our study was limited (no more than 3 years) and a few patients have occurred with clinical outcomes; 5-year. or even 10-year follow-up is required to further clarify the clinical benefit of using WFO and to provide more substantial evidence as to whether the cognitive computing system could be used as a clinical assistant to help physicians in making medical decisions. Finally, with the update of the NCCN guidelines and the accumulation of ou clinical experience, a blind trial may also need to be conducted.

## Conclusion

The recommended treatment regimens in patients with colorectal cancer were mostly concordant between WFO and MDT, with a concordance rate of 66.9%. We first found prognosis was better among patients in the concordant group than that in the non-concordant group, and especially that of “recommended” group was better than that of “not recommended” group. This study also presents a nomogram that incorporates tumor type, and the TNM stage, which can be conveniently used for individualized prediction of concordance, and can provide a useful tool for assisting physicians in making clinical decisions. However, our findings need to be prospectively validated in larger multi-center trials with long follow-up periods.

## Data Availability Statement

The original contributions presented in the study are included in the article/**Supplementary Material**, further inquiries can be directed to the corresponding author/s.

## Ethics Statement

The study was approved by the ethics committees of the Second Affiliated Hospital of Wenzhou Medical University, and all participants provided written informed consent prior to study participation, in accordance with the tenets of the Declaration of Helsinki.

## Author Contributions

XY and YY contributed to the data collection. CZ and JX contributed to the analysis writing. CM and XY contributed to the editing and submission of the article. XS and YH contributed to the conception of the project and editing of the article.

## Funding

This study was funded by the National Natural Science Foundation of China (grant no.81602165), the Zhejiang Medical and Health Science and Technology project (grant no. 2019317606), Zhejiang Public Welfare Technology Tesearch plan/social development project (grant no. LGF20H070003), and the Wenzhou Basic Scientific Research Projects (grant no. Y20180064 and Y20190060).

## Conflict of Interest

The authors declare that the research was conducted in the absence of any commercial or financial relationships that could be construed as a potential conflict of interest.
